# Development of an electrohydraulic drive system for the vibrating conveyor belt of the peanut digger-inverter

**DOI:** 10.1371/journal.pone.0203300

**Published:** 2018-10-30

**Authors:** Evaldo Ferezin, Rouverson Pereira da Silva, Adão Felipe dos Santos, Cristiano Zerbato

**Affiliations:** 1 Faculty of Technology of Sertãozinho (FATEC), Sertãozinho, São Paulo, Brazil; 2 Department of Agricultural Engineering, Laboratory of Agricultural Machinery and Mechanization, São Paulo State University, Jaboticabal, São Paulo, Brazil; Istituto Italiano di Tecnologia Center for Micro BioRobotics, ITALY

## Abstract

Hydraulic systems are equipment widely used in stationary industrial equipment and in moving agricultural equipment and construction machines. Currently, the rotation of the vibrating conveyor belt of the peanut digger-inverter is triggered by the tractor power take-off. Considering that for the equipment to operate at its optimal, the tractor engine needs to work at low rotation, responsible for the rotation of the power take-off, this work aimed at developing an electro-hydraulic system able to transmit varying work rotations to the vibrating conveyor belt of the digger-inverter, regardless the rotation of the power take-off and the speed used in the tractor. The electro-hydraulic system uses the oil of the auxiliary hydraulic system (remote control) and electric power of the tractor battery. The rotation of the hydraulic motor that drives the vibrating conveyor axis is controlled by a proportional flow control valve while the direction of rotation is determined by an electro-hydraulic directional valve, controlled by a Vcontrol Personal Device Assistant controller installed in the tractor cab. The electro-hydraulic system developed can be used to control the rotation of the vibrating conveyor belt of the peanut digger-inverter since it meets the torque and power requirements necessary to move the vibrating conveyor belt, with the respective rotation control.

## Introduction

The harvesting of peanuts requires crop-specific machines, such as the digger-inverter, which can dig the peanut vines from the earth, shake the soil from the vines and deposit the vines in an inverted position so as the pods are exposed to drying in the field. This important step in the productive process reduces the water content in the pods so they can be collected in a subsequent operation.

In Brazil, when using such equipment, the fact that the vibrating conveyor (responsible for transporting the plants to the inverting roller) is activated by the tractor power take-off (PTO) is a limiting factor. The power resulting from the transformation of chemical into mechanical energy in the engine [1,2], is redistributed through the transmission systems, finally reaching the PTO and the driving wheels [3].

The PTO peanut digger-inverter requires the tractor engine to work at low speed to ensure optimal equipment usage [4]. Several authors have confirmed that engine rotation combined with technical characteristics of the mechanized set increases fuel consumption and reduces engine life [5–9]. Several authors have already shown the importance of working at the adequate rotation in the PTO, regardless of the operation to be performed, and working speed [10–13].

So far, the alternative proposed to solve these problems is to use the auxiliary hydraulic system of the tractor for the digger-inverter, since these have wide applications due to high sensitivity, precision, rigidity, speed, power, and control capacity [14]. In agricultural tractors, this system is also known as remote control valves (RCV). The operation principle of this energy transfer equipment is based on transmitting the force through the pressurized oil flow [15], which activates the equipment, without interfering with the rotation of the engine.

Therefore, considering that the optimization of the peanut digger-inverter operation requires the tractor to work at low engine speeds (lower PTO rotation), the objective of this study was to develop an electrohydraulic system able to transmit varying working rotations to the equipment vibrating conveyor belt, regardless of the PTO rotation and tractor displacement speed.

## Material and methods

### System development and components

One of the most important components of the hydraulic system is the hydraulic pump that pumps oil in and out, leading to increased pressure when the outlet is blocked. The volume of oil carried per pump revolution (cm^3^ rev^-1^) under ideal conditions is called displacement. In an agricultural tractor, the oil pump used in the hydraulic system is driven directly by the motor rotation, and the maximum oil displacement occurs when the tractor is working at its nominal speed.

**Fig 1:**
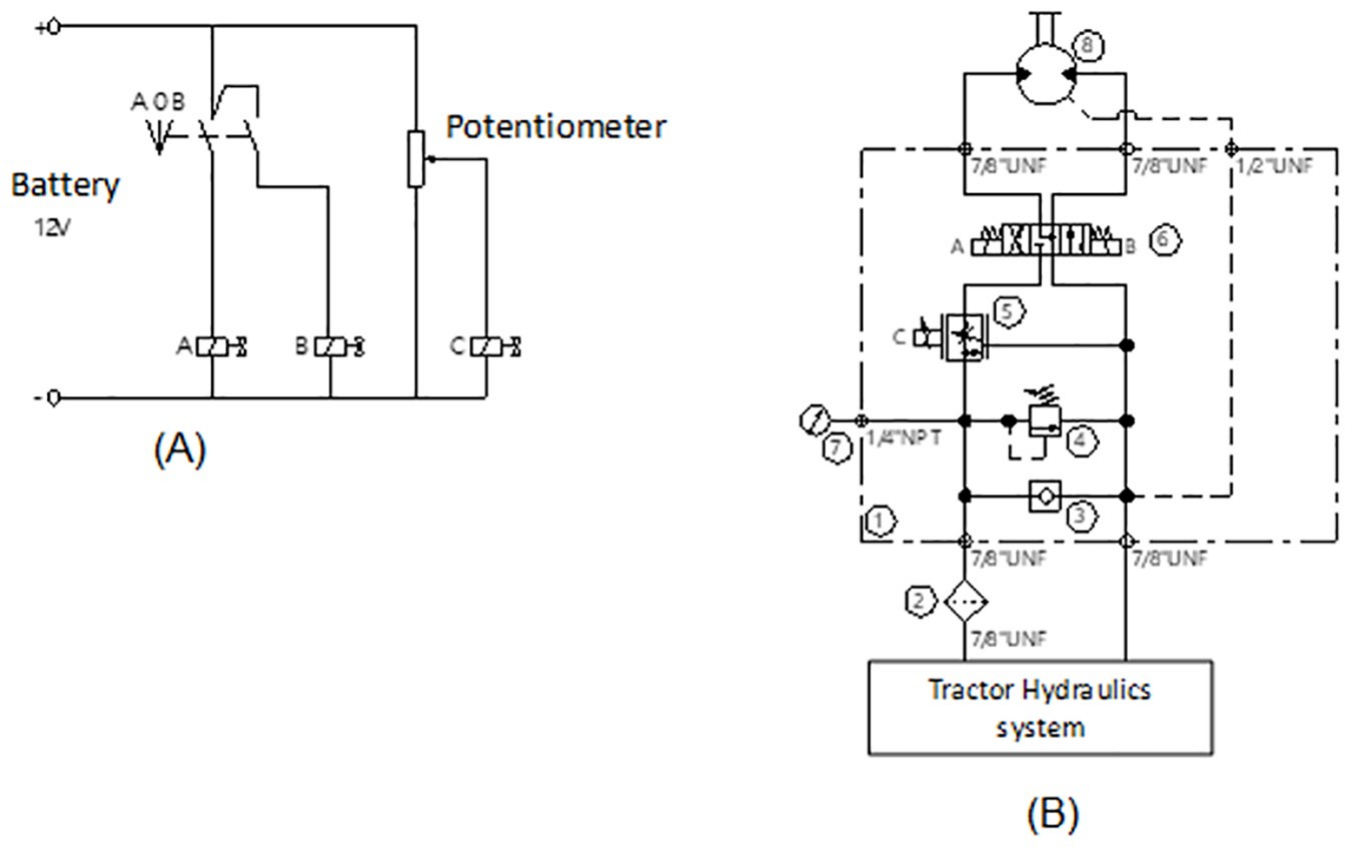
The electrohydraulic system driving the vibrating conveyor belt of the peanut digger-inverter: a) electric circuit; B) electrohydraulic circuit. (1) Manifold*; (2) Hydraulic filter; (3) Check valve; (4) Pressure regulating valve (5) Proportional flow control valve; (6) Directional valve; (7) Pressure gauge; (8) Hydraulic motor. *Manifold is a set of valves mounted in a single block.

Based on these considerations, an electrohydraulic system connected to the auxiliary hydraulic system of the tractor (remote control valves—RCV, was developed to maintain the conveyor-belt rotation by activating a hydraulic motor, acting independently of the PTO rotation. The solenoid valves allow controlling the rotation of the vibrating conveyor belt from inside the tractor cabin (Fig 1).

The tractor hydraulic system was used while the hydraulic control and the dynamic behavior were not considered in the design since the dynamic modeling of the system is not part of the objective of this work.

The auxiliary hydraulic system of the tractor supplies the hydraulic oil used to regulate the rotation of the vibrating conveyor-belt of the peanut digger-inverter.

### Dimensioning the hydraulic motor

From the design viewpoint, hydraulic motors are similar to pumps, and the theoretical flow of the engine, due to its displacement, is defined by the volume of oil transported by rotation, under ideal conditions, not including friction loss or leaks (Eq 1). Thus:

$$Q=\frac{D∙n}{1000}$$ (1)

here:

Q = theoretical flow (L min^-1^);

D = displacement (cm^3^ rev^-1^);

n = rotation (rpm);

1000 = unit conversion factor.

The theoretical torque of an ideal hydraulic motor is given by Eq 2:

$$T=\frac{\Delta P∙D}{2\pi ∙100}$$ (2)

Where:

T = theoretical torque (kgf m);

ΔP = pressure change (kgf cm^-2^);

100 = unit conversion factor.

The theoretical power of an ideal hydraulic motor, disregarding the power loss of the hydraulic fluid entering the engine, can be written by Eq 3:

$$N=\frac{n∙T}{729}∙735,75$$ (3)

Where:

N = theoretical power (W)

The hydraulic motor was dimensioned based on the rotation, the power and torque required to start and the pressure and flow of the hydraulic circuit oil.

The power required to transport the peanut mass on the vibrating conveyor belt is calculated by Eq 4:

$$N=\frac{M_{t}∙\pi ∙n}{30}$$ (4)

Where:

M_t_ = torque (Nm);

30 = unit conversion factor.

The torque required to transport the peanut mass on the vibrating conveyor belt is calculated by Eq (5):

$$M_{t}=F∙r$$ (5)

Where:

F = Tangential force caused by the load (N);

r = radius (m);

The tangential force on the vibrating conveyor belt of the peanut digger-inverter is given by Eq 6:

$$F=\frac{\emptyset ∙g}{v}∙c∙\text{cos}\;\theta$$ (6)

Where:

∅ = Mass flow of material on the vibrating conveyor belt (kg s^-1^);

g = gravity (m s^-2^);

v = Tangential velocity of the vibrating conveyor-belt (m s^-1^);

c = Wheelbase of the vibrating conveyor-belt (m);

∅ = tilt angle of the vibrating conveyor-belt (°).

By definition, in Engineering, mass flow is the amount of material that passes per unit area, per time (kg m^-2^ s^-1^). However, to suit the nomenclature used in Agronomy, in this work the material discharge (kg s^-1^) was treated as mass flow.

Finally, since the tangential velocity of the vibrating conveyor belt can be described by Eq (7), we have:

$$v=\frac{\pi ∙r∙n}{30}$$ (7)

**Fig 2:**
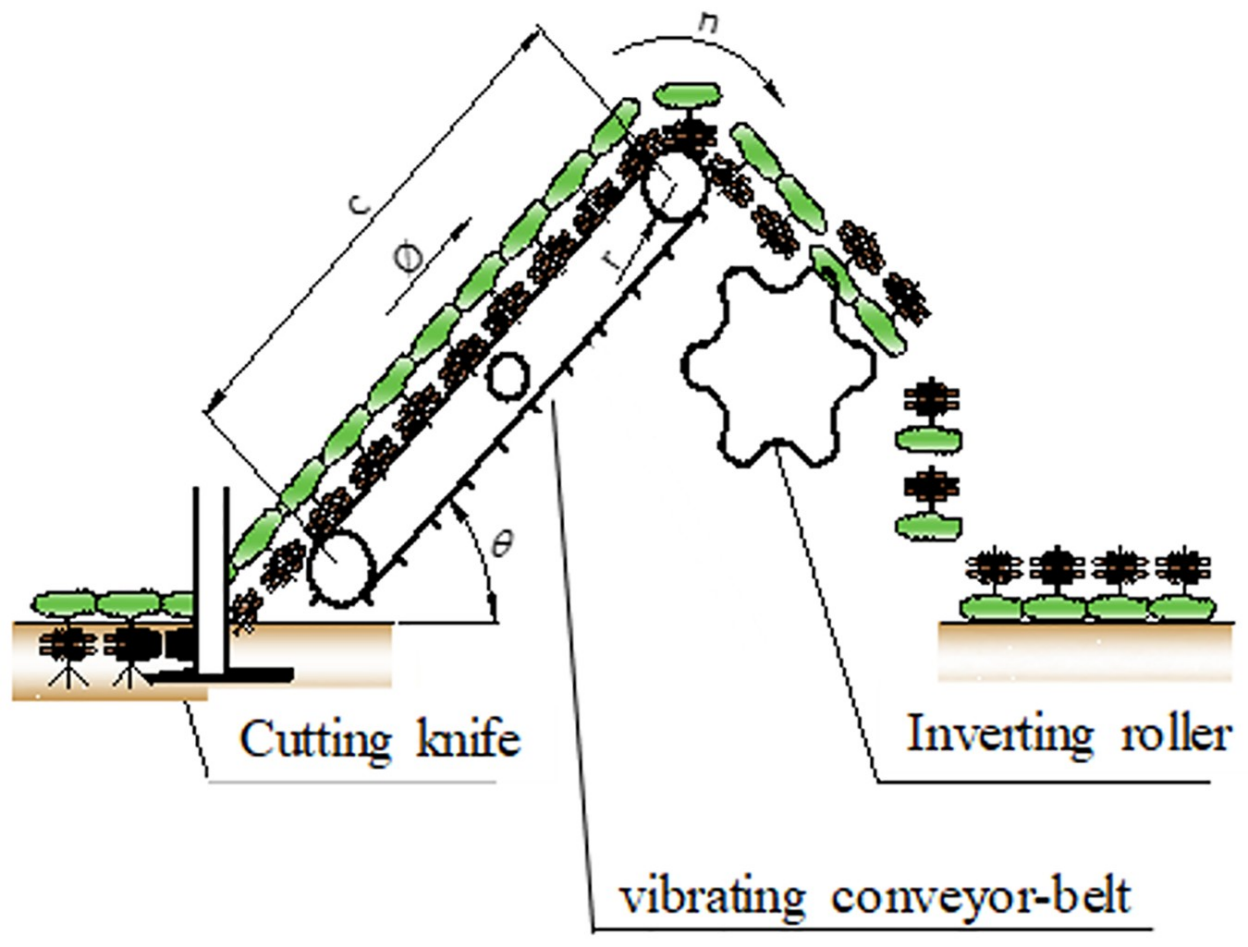
Schematics of the peanut digger-inverter. Knives cut the roots of plants to a depth of up to 15 cm. The plants are directed to the digger-shaker vibrating conveyor belt, then pass through inverting rollers that reverse the plants (leaving roots up and leaves down).

Where:

v = Tangential velocity of the vibrating conveyor-belt (m s^-1^);

r = radius (m);

n = rotation (rpm);

Thus, the hydraulic engine needed to drive this machine was calculated based on the 2-Row Model of peanut digger-inverter shown in Fig 2, and in the parameters of the vibrating conveyor belt (Table 1).

**Table 1:** Vibratory conveyor belt data used to dimension the hydraulic motor.

Description	Variable	Valor	Unit
Mass flow of material on the vibrating conveyor belt	∅	7.4	kg s^-1^
Axis radius of vibratory conveyor belt	*r*	0.1	m
Wheelbase of the vibrating conveyor belt	*c*	1.35	m
Tilt angle of vibrating conveyor belt	*θ*	45	°
Lower rotation of the vibrating conveyor belt axis	*n*	80	rpm
Higher rotation of the vibrating conveyor belt axis	*n*	120	rpm
Torque required to start the vibrating axis	*M*_*te*_	18.5	Nm

The calculations were based on the lower rotation of the vibrating conveyor belt because it requires a higher torque.

### Description of system operation

The description of the electrohydraulic system operation is based on Fig 1a and 1b, and the numbers below, in parentheses, refer to the components of the system.

The oil supplied by the tractor remote control is filtered to remove impurities and transfer to the manifold equipped with a proportional flow control valve (5), a pressure regulating valve (4), a directional valve (6) and a pressure gauge (7).

First, the hydraulic oil enters the proportional flow control valve (5), which controls the conveyor-belt rotation by varying the oil flow sent to the hydraulic motor. Then, the oil flows through a 4-way directional control valve (6) with three positions. The valve positioned in the center sends the oil back to the reservoir. The other position advances the conveyor to discharge the dug peanut vines into the rollers which then invert the peanut vines to expose the pods. The last position reverses the conveyor-belt rotation, when necessary, especially when problems occur during digging (bushing).

The control valve directs the oil to the hydraulic motor (8) which, in the forward position, causes the conveyor-belt to rotate in the desired direction, adjusted according to the operation needs.

The electrohydraulic system works with the 12 V battery of the tractor while all solenoid control valves are controlled from inside the tractor cabin by a box with the electric circuit, potentiometer, and knob.

The 4/3 way directional control valve (6) is controlled by a 3-position knob that determines the rotation direction of the hydraulic motor. The motor rotation is controlled by the proportional flow control valve (5), which is started by the potentiometer that regulates the proportional opening of this valve. The 3-position knob and potentiometer are installed in the electric control box in the tractor cab.

The rotation of the hydraulic motor and the speed of the tractor are monitored by a Vcontrol PDA controller (Personal Device Assistant) by Verion Agriculture, which is also installed in the tractor cab. This PDA controls the rotation of the hydraulic motor automatically, acting on the solenoid valves of the electrohydraulic system. Therefore, as the tractor speed changes, the conveyor-belt speed can also be changed.

In this system, the rotation of the vibrating conveyor-belt of the peanut digger-inverter is independent of the rotation of the tractor power take-off, and thus, the tractor can operate in the normal working rotation of the motor. The system works manually, using the knob and potentiometer, or automatically with the PDA.

### Exploratory tests

Exploratory tests are preliminary experiments to verify the behavior and efficiency of the equipment used. The following tests were carried out in this research:

### Software simulation test

The construction of the Electrohydraulic system model was performed at the Automation Studio^™^ P6 software by selecting the items of interest in the library. After the selection of all the items of the model, the connection was made to the same, in which the lines represent the pipes and the numbers the components of the system to be simulated (Fig 1). The software allows you to verify that all items are connected correctly and then you can choose many operating parameters for each item.

The hydraulic system simulation test in software is performed to analyze the behavior of the hydraulic system responsible for controlling the rotation of the vibrating conveyor belt before the bench simulation test. Thus, starting from the test results of fields presented by [4], were used for the simulation in software rotations ranging between 85 and 145 rpm, with increments of 15 rpm.

### Bench scale test

The objective of the bench simulation test of the electrohydraulic system was to analyze the behavior of the electrohydraulic system responsible for controlling the vibrating conveyor belt rotation before the components were purchased.

**Fig 3:**
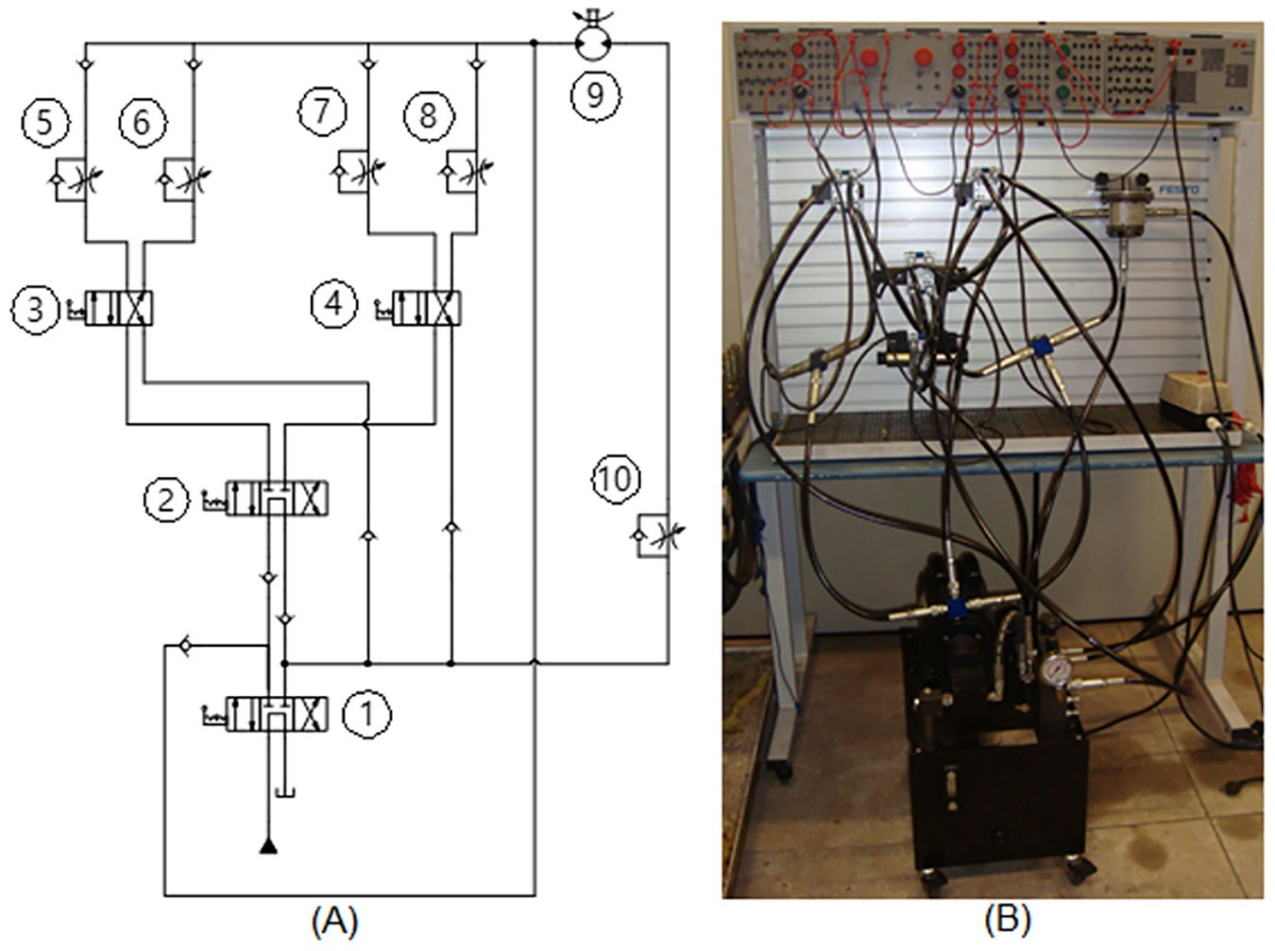
Schematics of the operation of the electrohydraulic system used in the test (a), and electrohydraulic circuit for simulating different rotations (b). (1 and 2): 4/3 way directional valve 5, 6, 7, 8; (3 and 4): 4/2 way directional valve; (9): Hydraulic motor and (10): Flow Regulating Valve.

The bench test was performed using the same hydraulic circuit assembled in the software simulation using two lever-operated directional valves and two solenoid electrohydraulic directional valves (Fig 3).

### Field test

The experimental area used was approximately 1 ha, located near the 21°14’S and 48°16’W geodesic coordinates, in Jaboticabal, São Paulo, Brazil, at 560 m average altitude, and 4% average slope. According to Köppen, the climate is classified as Aw (subtropical). The soil of the experimental area is typical Eutroferric RED LATOSOL with clay texture [16].

No specific permissions were needed to access the experimental sites because all activities were performed under the supervision of the advisor of the thesis and did not involve threatened or protected species.

The soil was conventionally prepared with a plow (disc plow) and three harrows (one heavy and two lights). The cultivar Runner IAC 886 (*Arachis hypogea* L.) was sown using a pneumatic sowing-fertilizing machine with 0.9 m spacing between rows, at sowing density of 18 seeds m^-1^.

For the purposes of characterizing the harvesting conditions, the water content of the soil and the pods were sampled [17], the productivity and ripening of the pods [18], which presented average values of 26.7%, 55.5%, 2432 kg ha^-1^ and 74.4%, respectively.

The digging operation was performed using a 2 rows model digger-starter-shaker, model C 200 (MIAC—Pindorama, São Paulo, Brazil), with 1.8 m working width, pulled by a Massey Ferguson MF 718 tractor, 4x2 TDA, with 132.4 kW (180 hp) power at 2200 rpm. The flow rate and maximum oil pressure of the hydraulic system of this tractor are 138 L min^-1^ and 20 MPa, respectively. The operation was carried out in the working gears H1 (3.5 km h^-1^), L2 (4.2 km h^-1^) and H2 (5.0 km h^-1^). These speeds were selected because they are commonly used in fields in PTO diggers [19].

The experimental design consisted of testing 5 different rotations of the vibrating conveyor-belt of the peanut digger-inverter (80, 90, 100, 110 and 120 rpm) and 3 displacement speeds of the machine/tractor set (3.5, 4.2 and 5.0 km h^-1^). The tests were performed with 3 replicates.

The data were collected in 2 rows of peanuts, forming 1 windrow for each speed and rotation, in 40m-long sample plots, keeping the 50m distance of the carriers, for stabilizing the machine/tractor set.

The crop-related variables (visible, invisible and total losses in the digger), and the mechanized set variables (height and width of the formed windrow after digging) were analyzed to evaluate the performance of the peanut digger-inverter. The losses were evaluated according to [20] and calculate based in yield media.

Analysis of variance (ANOVA) was performed with a block randomized analysis using AGROESTAT software. Differences between treatments were analyzed using test t students of one factor ANOVA at the 5% probability level. When the value of the F test was significative to interaction among the factors, was analyzed by means of regression, opting for the adjustment of higher value of R^2^.

## Results and discussion

### Software simulation test

The simulation in the software allowed to verify which and how many components would be needed for the system to work in the next test step, allowing them to be defined as required components Two 4/3-way directional valves, two directional valves 4/2 via and four flow regulating valves, plus a hydraulic motor. The simulation allowed to gauge that these components would be necessary and sufficient for the proper functioning of the system on the test bench.

The description of the operation of the electrohydraulic system is based on Fig 3, and the numbers below, in parentheses, refer to the components of the system.

For this test, the engine speed was regulated by varying the opening of the flow control valves placed in the hydraulic motor supply lines.

The directional valve (1) represents the remote-control valve of the tractor, which when activated releases oil to the hydraulic system allowing the hydraulic motor (9) to move the vibrating conveyor belt either forward or backward, depending on the valve position.

If the valve (1) is positioned to the right, the released oil moves the hydraulic motor forward (9), and by turning the directional valve (2) to the right, the oil is sent to the directional valve (3). If the valve (3) is positioned to the left, the hydraulic oil passes through the flow control valve (5) to obtain the first rotation of the hydraulic motor. If valve (3) is positioned to the right, the hydraulic oil is fed to the flow regulating valve (6) to obtain the second rotation of the hydraulic motor.

If the directional valve (2) is positioned to the left, the hydraulic fluid is sent to the directional valve (4), which turned to the right position determines the third rotation in the hydraulic motor and the oil is transported to the flow regulating valve (7). However, if valve (4) is turned to the left, the fourth rotation of the hydraulic motor is achieved with the hydraulic oil passing through the flow regulating valve (8).

By turning the directional valve (1) to the left position, the hydraulic oil is directed to the flow regulating valve (10) and the hydraulic motor (9) turns in the opposite direction (reversing the direction of the vibrating conveyor belt).

### Bench test

After the simulation on the software the system was mounted in the bench test, from the components defined in the computational simulation, considering a manual drive with an inlet flow rate of 60 L min^-1^ and displacement of 125.7 cm^3^ rev^-1^ (Table 2).

**Table 2:** Results obtained with the hydraulic system bench test in simulation software.

Q (L min^-1^)	10,68	12,57	14,46	16,34
ղ (rpm)	85	100	115	130

This test showed that it is possible to vary the rotation of the hydraulic motor by simulating four different pre-established rotations of the vibrating conveyor belt of the digger-inverter and change the rotation direction. The inversion can be used in the event of the conveyor-belt stopping due to possible overload of the hydraulic motor.

Similarly, [21] presented the design and simulation of an automatic shift-speed controller of the sugar cane harvester using the hydraulic and mechanical parameters of the harvester in the simulations. The authors concluded that the controllers can incorporate managerial strategies making the operation of the more appropriate harvest for each situation and relieving the work of the operator. [22] they adapted a productivity monitor with impact card for peanut culture, with two different mounting configurations, one floating and one hinged, obtaining an average error of 12.7% in the floating configuration and 6.6% in the articulated configuration.

Aiming at improvements in the peanut harvest, [23] developed a depth control system from digging to a peanut digger-shaker-inverter, controlling the position of the system hydraulic three-point, to reduce peanut losses to different types of soil. The equipment was tested by [24] in three types of texture of different soil (Sandy, medium and clay), which concluded that the prototype developed demonstrated the potential in reducing losses of $47 ha^-1^. In this way, it is assumed that in the field, the electrohydraulic system can contribute to greater control of the quality in the operation of digging, giving greater autonomy in the performance of the starter-shaker-inverter.

Also, the test bench simulation allowed to verify that it was possible to reduce the number of components of the system by replacing the directional valves and flow regulators with a proportional direction valve. This proportional directional valve is a solenoid valve that allows the variation of rotation in the desired range, regulating the oil flow with infinite increments. In this way, the voltage increases provide small variations in the flow of the hydraulic oil, allowing the desired change of rotation.

### Field test

Based on the results of the field using digger-shake-inverter with electrohydraulic system, it was observed that visible losses were influenced by the interaction of the shifting speed and the rotational increase of the conveyor belt in all the tested combinations, except when the mechanized set (tractor-digger) displaced at the speed of 4.2 km h^-1^.

The digger-shaker did not statistically influence the unseen losses during the peanut digging (data not shown). This result was to be expected, since the invisible losses are not related to the drive system of the starter, and the occurrence of such losses is related to factors such as soil type, soil water content and maturation, which can act in isolation or even together [25–26].

**Fig 4:**
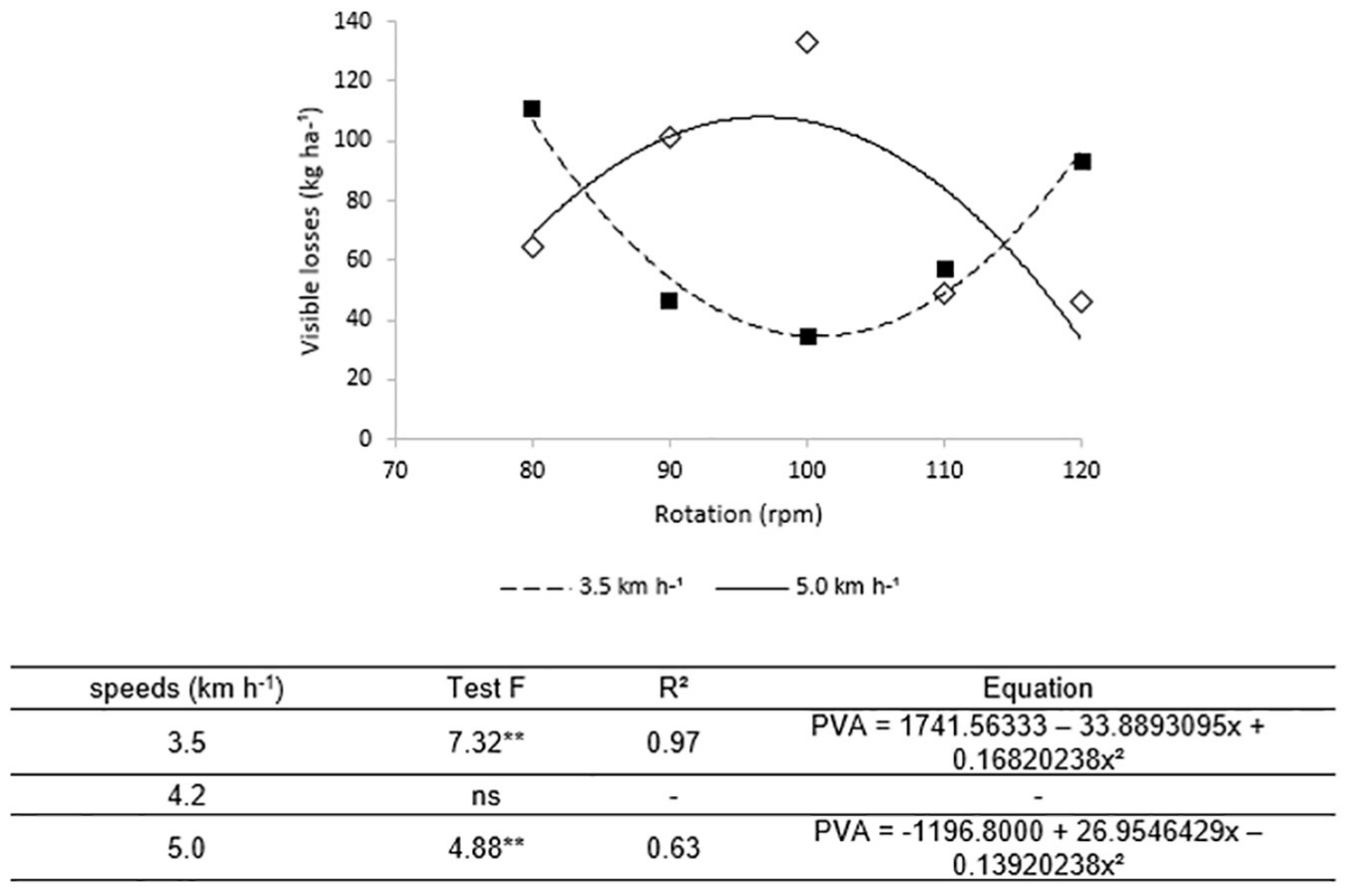
Estimated visible losses versus the rotation of the vibrating conveyor belt for the velocities 3.5 and 5 km h^-1^. The equation of adjustment of each interaction is shown below Fig 4, when this was significative by the F test (p<0.05). The speed 4.2 km h^-1^ did not obtain significant adjustment (ns).

The digging visible losses (Fig 4) for the 3.5 km h^-1^ velocity shows a convex curve, indicating greater losses in the lowest and highest rotations investigated while the lowest loss was observed at the inflection point, approximately 110 rpm. The curve behavior shows that digging at lower and higher rotations increased the losses while the highest losses were observed at the lowest rotation (80 rpm). Therefore, to minimize the digger visible losses for this velocity, the optimal work rotation of the conveyor is between 100 and 120 rpm with the digger-inverter performance peaking at 110 rpm.

However, it is observed that when it was used 100 rpm in the rotation of the conveyor belt there was significant increase of the visible losses, so that as it increased the shifting speed also increased the losses (34.63 kg ha^-1^ and 132.96 kg ha^-1^ at a speed of 3.5 km h^-1^ and 5.0 km h^-1^, respectively). These results indicate that when the Electrohydraulic system digger-shaker performs the digging operation at low speeds, it is recommended to use the conveyor belt rotation at 100 rpm.

The field tests using the Electrohydraulic model starter also made it possible to check, in general, that independent of the displacement speed of the tractor-digger set, the use of the smallest rotations in the vibratory mat of the digger-shaker was where the highest levels of losses were obtained.

This fact is mainly due to the increase of material harvested by the digger-shaker under the conveyor belt and because it is in low rotation cannot perform the process of reversal of the plants. Thus, the plants are for a longer period under the vibration process of the conveyor belt, and therefore the detachment of the pods of the plants, mainly of the pods with high maturation level [27]. In this case, it cannot be said that the losses are directly related to the amount of plant mass the vibrating conveyor belt, as observed by [28], which report that the increase in the plant mass in the vibratory mat cushions the impact of vibration reducing the take-off of the pods.

For the mechanized set displacement velocity of 5 km h^-1^, the losses remained high at the lowest rotations up to 110 rpm, with a tendency to decrease at the higher rotations, which can be observed by the curve behavior and the losses at 120 rpm. This result indicates that 120 rpm is the most suitable at this speed, from the viewpoint of digging losses (Fig 4).

When it takes into consideration that peanut crops do not have uniformity in the production and quantity of plant matter, it indicates that the conveyor belt rotation must be adjusted according to the tractor’s displacement speed, however, this adjustment must be carried out throughout the operation of digging and taking into consideration the plant-machine relationship.

In work prior to the development of the electrohydraulic system proposed in this work and using similar displacement speeds, it was verified that, when the digger-inverter is triggered by the PTO, it also gets high levels of losses [26,28–29] and stop for maintenance due to the increase in plant mass in the vibratory conveyor belt.

It is worth pointing out that the drive diggers by the PTO require a rotation of approximately 350 rpm in the TDP so that there is good quality in the process of reversing the plants [4], these do not have the reversal mechanism, in case of excess vegetable mass on the mat, which increases the stop for maintenance. However, this rotation indicated by the manufacturer of the diggers is below the limit set by the technical standards for agricultural machinery, which is 540 rpm, and has a rotating movement only to one side.

On the other hand, the drive of the digger-inverter by means of the electrohydraulic system developed in this work, allows the digger-inverter to be triggered independently of the motor rotation of the tractor, because it has its own system of drive and the possibility of reversing the motion of the vibrating conveyor belt, as described in the methodology. In addition, the digging operation can be performed with higher displacement speeds than the conventional (up to 5 km h^-1^), for higher values than that, new field tests are needed to verify the efficiency of the equipment under these conditions.

As important as the losses, the dimensional characteristics of the windrow (height and width) formed after the process of the digger-inverter are fundamental to the success of the gathering of pods, subsequent operation that occurs three days after the digging.

**Fig 5:**
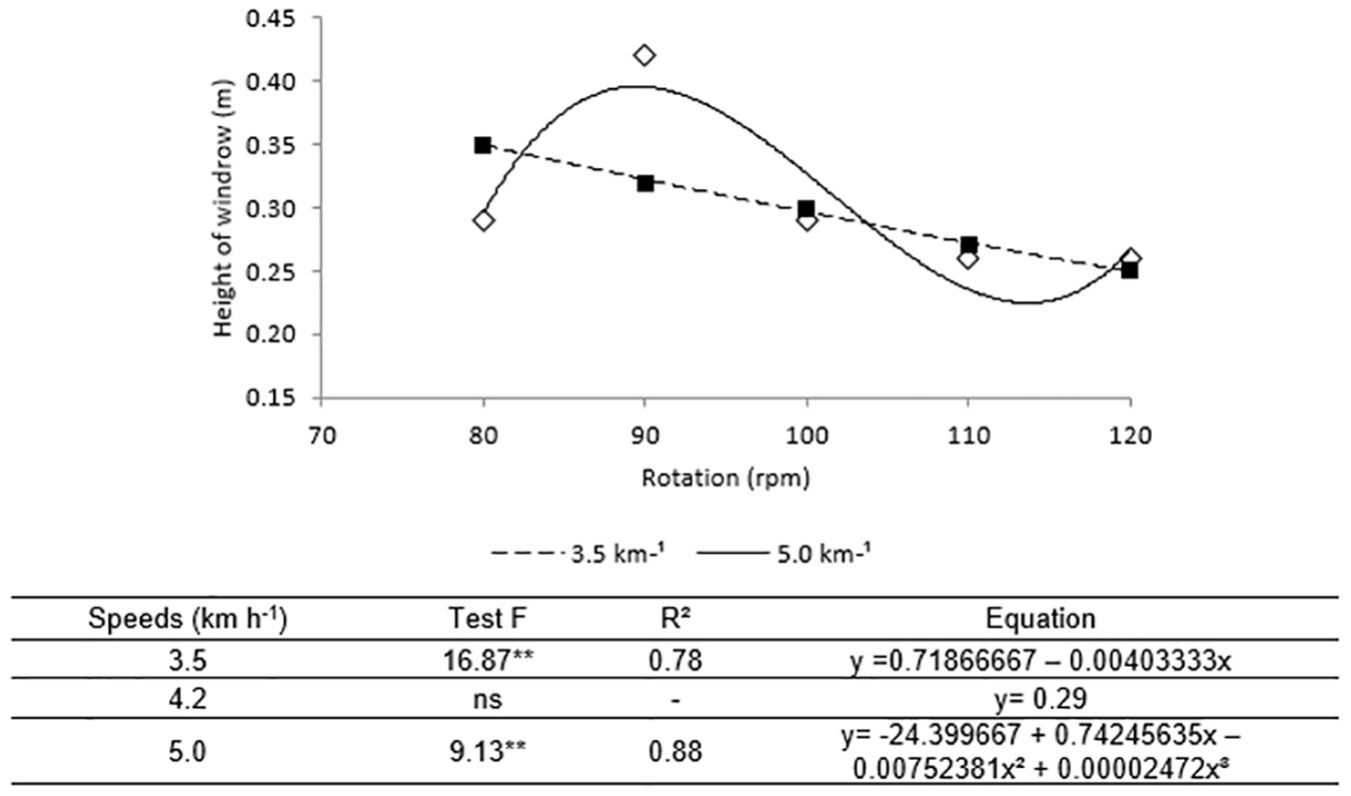
Windrow height versus rotation of the vibrating conveyor belt for the velocities 3.5 e 5.0 km^-1^. The equation of adjustment of each interaction is shown below Fig 5, when this was significative by the F test (**p<0.05). The speed 4.2 km h^-1^ did not obtain significant adjustment (ns).

The use of the smallest conveyor belt rotations (80 and 90 rpm), were the ones that provided greater height and width of the windrow, when it used the speeds of 3.5 and 5 km h^-1^ (Fig 5). In so far as increase was made in the rotation of the conveyor belt the height of the windrow decreased at these speeds, moreover, the shifting speed of the mechanized assembly interfered in this characteristic, reducing the height of the windrow as it increased the speed at the slightest rotation and in reverse rotation of 90 rpm.

Despite the instability in maintaining near average values, with a relatively large oscillation in the smallest rotations when the mechanized set shifted at the speed of 5.0 km h^-1^ (Fig 5), a certain uniformity is noted at the time of windrow in the rotations above 100 rpm, confirmed by the T test. This uniformity in the distribution of plants is interesting because it shows that the starter can work with a range of rotations without losing quality in the operation. When the digger-inverter performs the digging forming uniform windrows, the variability in the losses in the gathering operation tends to be lower [29], Which consequently optimizes the efficiency of the harvester.

**Fig 6:**
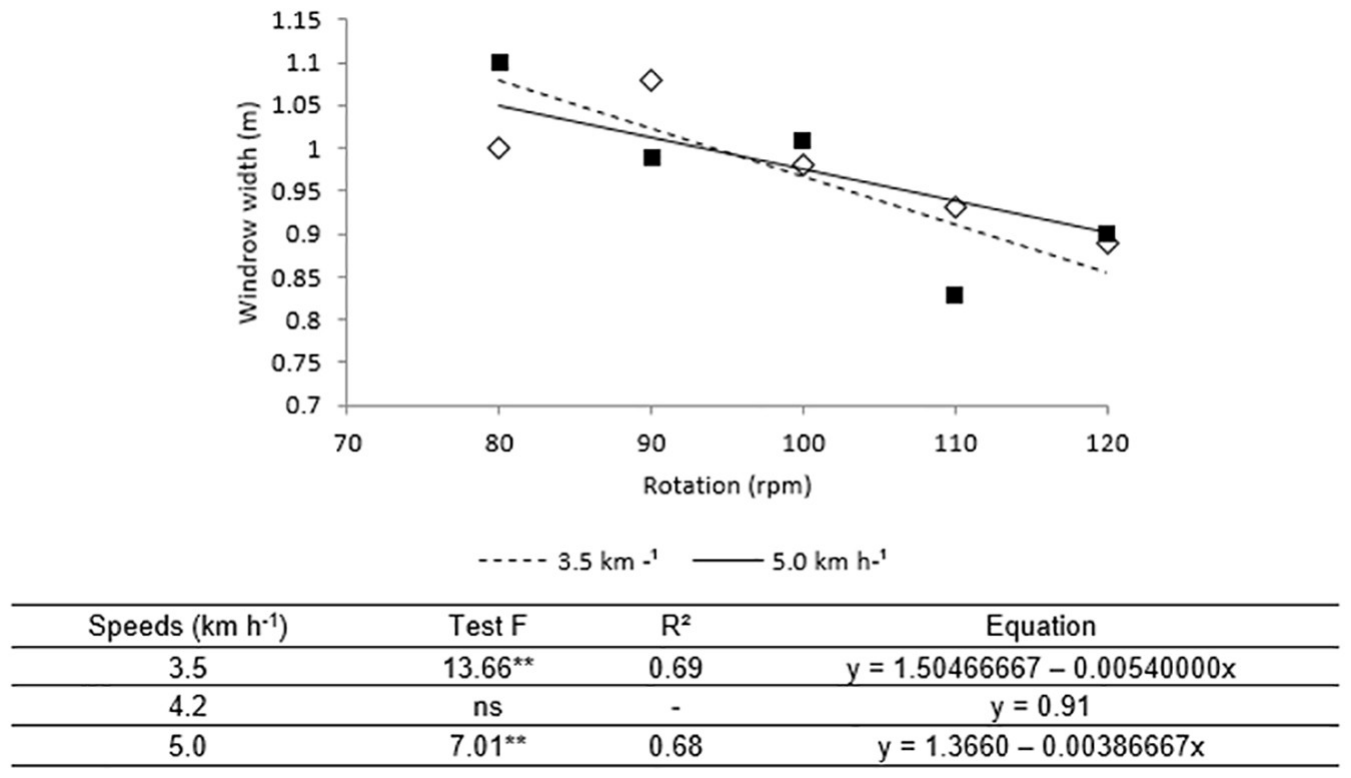
Windrow width versus conveyor belt rotation after the passage of the mechanized set at the velocities. The equation of adjustment of each interaction is shown below Fig 6, when this was significative by the F test (**p<0.05). The speed 4.2 km h^-1^ did not obtain significant adjustment (ns).

In relation to the width of the windrow it was observed that the highest rotations independent of the speed of displacement provided windrow less wide (Fig 6). The effect of the shift speed interfered only when the operation was performed with the rotation of 90 rpm, providing wider windrow when the operation was performed at the highest and lowest speed.

The width of the windrow does not depend solely on the rotation of the vibrating conveyor belt, since it is the correct adjustment of the fingers queuing on the back of the digger that gives the format to the windrow, mainly width. However, adjustment of the queued fingers has been maintained for all field test conditions. Thus, the results found lead us to infer that the higher the conveyor belt rotation, the greater the width of the windrow and the lower the height. The higher rotations may have thrown the plants farther, causing these results. However, there is no account in the literature of ideal dimensions for the windrow formed after the arranquio of peanuts.

When does not have uniformity in the distribution of windrow, with points in Isolated from piled up of the plants can increase the variability of losses and reduce the quality of the harvesting operation, as it tends to increase the flow of material in the machine gathering platform, which can increase the losses in the platform of feeding, causing losses even before the material passes in the system of track and separation of the machine [29].

In this way, it is essential that the operator of the tractor-digger set attend to the rotation of the vibrating mat of the starter, adjusting according to the need of the operation. It is emphasized that using the electrohydraulic system, it is possible to adjust the inside of the tractor cabin.

## Conclusions

The developed electrohydraulic system is safe since it can avoid the equipment to break in the presence of obstacles by controlling the rotation of the vibrating conveyor belt and the inverting rollers, regardless of the PTO. Also, this system can be operated automatically by varying the rotations according to the tractor displacement speed, allowing the best adjustment of the rotation of the digger-inverter vibrating conveyor belt to the different conditions encountered during the peanut digging operation.

The evaluated equipment can work at 5.0 km h^-1^ displacement speed, leading to greater operational field capacity while the losses remain close to those obtained at 3.5 km h^-1^ speed.

During the field test, the digger-inverter presented neither embedding nor any other problems, showing that the machine can be used efficiently in the mechanized peanut digging. Also, because it works independently of the PTO, it improves the tractor performance, which can then operate at the nominal engine speed, reducing specific fuel consumption and increasing the power available for the mechanized set.

## Supporting information

S1 Data File [Data_field_digger.xlsx]. (XLSX)
